# Desiderata for digital consent in genomic research

**DOI:** 10.1007/s12687-017-0355-z

**Published:** 2018-01-23

**Authors:** Carlos Luis Parra-Calderón, Jane Kaye, Alberto Moreno-Conde, Harriet Teare, Francisco Nuñez-Benjumea

**Affiliations:** 1 Group of Research and Innovation in Biomedical Informatics, Biomedical Engineering and Health Economy, Institute of Biomedicine of Seville, IBiS / Virgen del Rocío University Hospital / CSIC / University of Seville, 41013 Seville, Spain; 2 HeLEX Centre, Nuffield Department of Population Health, University of Oxford, Oxford, UK

## Abstract

Herein, we describe the characterization of a Digital Consent (DC) System to support current ethical-legal issues associated with challenges posed by informed consent for genomic research. A potential solution to support ongoing interaction with patients and allow control over how their data and samples are being used in genomic research can be Digital Consent based. But there are other challenges that need to be addressed, such as incidental findings when analyzing the results of genomic tests (not expected). This paper addresses security and privacy recommendations for the development of precision medicine, and the interoperability references of Health Information Standardization Organizations such as HL7 and IHE, as well as recent research in the field of ethics in Genomic Medicine. As a result of this work, ten key features that need to be further explored have been identified in order to support the realization of DC in Genomic Research.

## Introduction

Advanced technologies and expanded research opportunities in genome medicine have posed a significant challenge to informed consent. For example, next-generation sequencing technologies (NGS) are being rapidly adopted in clinical research and routine care. In the case of genomic research, the biomedical research domain that makes use of genomic information, it is actually a challenge to make understandable for the research participant the results of the application of NGS technologies. In this sense, a robust informed consent process tailored to genomic research is required (Grady 2015). But this informed consent must also meet the requirements for genomic-based healthcare, clearly distinguishing the different roles (patient, research participant or both of them) that one subject may acquire.

This combination of roles, added to the rapidly evolving research methodologies and novel genomic testing procedures brings ethical uncertainty to genomic research. At present, we cannot anticipate or control the implications of these changes, which means that the research participant must be adequately informed about these results, or any changes, to continue to give appropriate informed consent.

These challenges also include some issues, like the inability to clinically address many of the findings of genomic testing, the degree of detail to include in informed consent processes, the application of new digital formats to obtain informed consent, and the expectations of patients and research participants (Adams and Petersen 2016).

Dynamic consent has been presented in the literature as a potential solution to support ongoing interaction with patients while providing them with greater control on how their data and samples are being used (Kaye et al. 2014; Budin-Ljøsne et al. 2017a). Digital technology is a significant part of this, as informatics can facilitate consent and monitor the use of data and samples. Furthermore, a semantically enriched dynamic consent would improve its adaptability to every new research scenario (Tenenbaum et al. 2016). This paper therefore considers some of the technical challenges related to implementing a digital dynamic consent.

Health Level 7 (HL7) and Integrated Health Enterprises (IHE) initiatives are examples of international efforts to standardize electronic consents. HL7 defines a semantically robust structure for the univocal computation of consents, including the privacy rules that apply to each case. HL7 is an international organization that defines and publishes specifications, protocols and standards to exchange information from different, dispersed and heterogeneous health information systems. Certified by ANSI since 1994, HL7 releases standards, applications, biomedical terminologies, clinical document designs, conceptual schemes, applications, and architectures on data exchange (Based et al. 2016). The IHE is an international voluntary collaboration of vendors, healthcare providers, regulatory agencies, and independent experts working on improving medical data interoperability in specifics domains, by generating integration profiles, which are detailed requirements of the application of existing standards such as HL7 itself, in the management of specifics use cases of clinical and health information management and exchange.

The IHE organization is currently working on the definition about how to implement Digital Consent in IT infrastructures. This organization defined the Basic Patient Privacy Consent Profile, which shows how to capture a patient’s acknowledgment and/or signature for one or more of these policies. The Advanced Patient Privacy Consent allows for the transport of a structured policy representation using a consent document. This can be used by an unspecified enforcement mechanism (e.g., within an existing access control system) to perform automated access checks ().

This desiderata proposal provides an initial list of issues to be considered, to initiate discussion about additional features needed to ensure that Digital Consent is a suitable tool for overcoming the described challenges.

## Desiderata

Some of the features that have emerged from recent discussions relating to consent in genomic research include:

1. Consent management: secure, continuous and patient-controlled consent management, relating to the study and sample tracking. This will include the development of structured consent forms and the adoption of relevant ontologies to represent the semantic relationships of regarded concepts and terms. User interface design and technological infrastructure will need to enable continued participant engagement after the point of enrolment. This feature will allow participants to be automatically updated when new research studies in need of analyzing their data are initiated (Tenenbaum et al. 2016; Williams et al. 2015).
2. Participant recruitment in Genomic Research: where appropriate, online participant recruitment should allow a personalized proposal for the participation of subjects in research studies, clearly explaining how potential study candidates have been identified, with adequate references, well-identified risks and opportunities to contact the study team (Budin-Ljøsne et al. 2017b). In many instances, face to face recruitment will remain the most appropriate method.
3. Patient and Research Participant Information Services: Both patients and research participants must be able to monitor and keep track of each researcher’s access to their data sets and biological samples over time, including accessing research outcomes, highlighting those where their data or biological samples have been used (Maxwell et al. 2016).
4. Integration in the Genomic Research and Healthcare processes: Consent must be integrated for research and healthcare, mainly as a requirement for the implementation of Learning Health Systems, in which research and healthcare activities are carried out simultaneously, supporting the integration of information derived from patient care and research, enhancing evidence generation to efficiently integrate improved prevention, treatment, and care-delivery methods. An ethical-based learning health care system must have core commitments to engagement, transparency, and accountability in ways that are keenly sensitive to the rights and interests of patients (Grady 2015).
5. Healthcare professionals, patients and research participant literacy: this feature addresses the complexity of genomic information that must be improved to ensure that patients and research participants understand the implications of NGS technologies. This is particularly challenging given that advanced sequencing technologies are difficult to fully understand even for healthcare professionals. This feature is a great challenge for biomedical informatics, as patient understanding may be key to the successful application of precision medicine that refers to the tailoring of medical treatment to the individual characteristics of each patient (Grady 2015).
6. Incidental findings in Genomic Research: Consent must support the right to know or not know about incidental findings during genomic research (Kaye et al. 2014). Consent should also be able to limit access to specific information as determined by the patient’s consent decisions. This feature requires that data elements of consents must be expressed semantically and with sufficient granularity to be managed (Based et al. 2016; Maxwell et al. 2016).
7. Accessibility: Digital consent must be accessible from any device and interface, from all possible electronic forms. Consent data should be processed at different encryption levels depending on the risk grade of the device used to access data (Boutin et al. 2016; Maxwell et al. 2016).
8. Identity management: consent must provide a trusted identity management process, including identity proofing, credentialing, authentication and authorization (Maxwell et al. 2016).
9. Audit trail: audit must be available to reliably track consent status, including records of each consent transaction over time. This should also include the identification of research users accessing consent information and their functions (researchers, biobanks technicians, etc.) (Budin-Ljøsne et al. 2017b; Maxwell et al. 2016).
10. Security and Privacy standardization: The concepts and rules of security and privacy associated with Digital Consent should be standardized, consistent and accurate to avoid ambiguity in their interpretation (Based et al. 2016).

## Discussion

This manuscript highlights some of the challenges that will need to be addressed in the near future in order to integrate consent in research and health care for the use of samples and biomedical data under the Health Learning System paradigm. It recognizes the need for explicit consent with precise and continuous information on each of the uses of patient information and biological samples in the scope of genomic research.

The identified features relate to the different actors involved, the nature of the information, the need for access limitation and the importance of trust and therefore transparency when using patient data for research.

In response to the introduction of the European Data Protection Regulation is expected that consent management systems will need to be upgraded, and that digital systems will allow consent to be tracked and supported more easily. The establishment of a prioritized research agenda associated with the management of patient consent for genomic information highlights existing needs not covered by the certified Electronic Health Records systems, and will help to coordinate R&D activities.

## Conclusion

This manuscript aims to guide discussions relating to future challenges associated with the implementation of DC in IT infrastructures, based on the comparison between those technologies applied in the certification process of eHealth systems. The comparison performed identified that challenges associated with the implementation of DC in the genomic field were not fully addressed by existing generic Digital Consent initiatives like IHE profiles. It is relevant to highlight these limitations to guide the technology evolution towards the implementation of appropriate mechanisms for DC management. This will help to address new challenges associated with the incorporation of personalized medicine in healthcare systems.
